# Association between Occupational Exposure to Wood Dust and Cancer: A Systematic Review and Meta-Analysis

**DOI:** 10.1371/journal.pone.0133024

**Published:** 2015-07-20

**Authors:** Montserrat Alonso-Sardón, Antonio-J. Chamorro, Ignacio Hernández-García, Helena Iglesias-de-Sena, Helena Martín-Rodero, Cristian Herrera, Miguel Marcos, José Antonio Mirón-Canelo

**Affiliations:** 1 Department of Preventive Medicine and Public Health, University of Salamanca, Institute of Biomedical Research of Salamanca, Salamanca, Spain; 2 Department of Medicine, University of Salamanca, Institute of Biomedical Research of Salamanca, Salamanca, Spain; 3 Department of Preventive Medicine, University Hospital Infanta Leonor- Virgen de la Torre, Madrid, Spain; 4 Department of Preventive Medicine and Public Health, University of Salamanca, Salamanca, Spain; 5 Biomedical Branch Library, Medical School, University of Salamanca, Salamanca, Spain; 6 Medical School, University of Salamanca, Salamanca, Spain; University of Kentucky, UNITED STATES

## Abstract

**Objective:**

To perform a systematic review to analyze the association between occupational exposure to wood dust and cancer.

**Methods:**

A systematic literature search of entries made in the MEDLINE-PubMed database between 1957 and 2013 was conducted to identify studies that had assessed the relationship between occupational exposure to wood dust and different types of cancer. A meta-analysis of selected case-control and cohort studies was subsequently performed.

**Results:**

A total of 114 studies were identified and 70 were selected for review. Of these, 42 studies focused on the relationship between wood dust and nasal cancer (n = 22), lung cancer (n = 11), and other types of cancer (n = 9). Low-to-moderate quality evidence that wood dust acts as a carcinogen was obtained, and a stronger association between wood dust and nasal adenocarcinoma was observed. A lesser association between wood dust exposure and lung cancer was also observed. Several studies suggested that there is a relationship between wood dust and the onset of other cancers, although there was no evidence to establish an association. A meta-analysis that included four case-controls studies showed that workers exposed to wood dust exhibited higher rates of nasal adenocarcinoma than other workers (odds ratio = 10.28; 95% confidence interval: 5.92 and 17.85; *P*<0,0001), although a large degree of heterogeneity was found.

**Conclusions:**

Low-to-moderate quality evidence supports a causal association between cancer and occupational exposure to wood dust, and this association was stronger for nasal adenocarcinoma than for lung cancer. There was no evidence of an association between wood dust exposure and the other cancers examined.

## Introduction

Dust generated in wood processing is one of the most common occupational and carcinogenic agents identified to date. The manipulation of wood can create fine and abundant dust with sanding, and thicker dust with milling or cutting [1,2,3]. The location and accumulation of particles has been found to depend on the size, shape, and density of the air flow available. Dust accumulates in the nose or the respiratory tract when the particles are larger or smaller than 5 microns, respectively [4,5].

Exposure to wood dust has been associated with several health problems, including pulmonary pathologies and other conditions [6,7]. In particular, cancer is a pathology that has been associated with wood dust [8–13]. Consequently, in 1995, the International Agency for Research of Cancer defined wood dust as a group I human carcinogenic substance [14].

Exposure to wood dust can vary considerably among populations, and it has not been found to be specific for a single sector or professional group, or for a single cancer. However, exposure to wood dust has been specifically linked to adenocarcinoma (ADCN). Currently, exposure to wood dust has a large impact on occupational health, and its occupational prevalence ranges from 10% to 15%. While occupational exposure to wood dust potentially contributes to an increased mortality rate for certain workers, it can also affect the mortality rate of the general population. Therefore, based on the health and social impacts of wood exposure, it is important to recognize this risk and to provide adequate professional and occupational protection.

Systematic reviews and/or meta-analyses represent useful methodological tools for assessing published data, and they also provide valid and reliable evidence for hypotheses [15,16]. Over the last few years, a consensus has been established to facilitate an assessment of the different primary studies that have been conducted, and to improve the quality and homogeneity of the systematic reviews that are conducted. With this in mind, it is appropriate and necessary to perform a systematic review that offers evidence on the relationship between different kinds of cancer and occupational exposure to wood dust.

Therefore, the aim of this systematic review was to analyze the data of previously published studies in relation to work exposure to wood dust and the onset of cancer.

## Materials and Methods

### Study design and selection criteria

A systematic review of the literature was conducted in order to identify studies that assessed the relationship and association between occupational exposure to wood dust and cancer. Selected articles (published in English or Spanish) that were available on Medline and included primary data collected between 1975 and September 2013 were selected. The exclusion criteria for this study ruled out works which consisted of opinions and/or recommendations from experts, as well as observational and experimental research studies.

### Search strategy

To perform the initial bibliographic search of the MEDLINE database, the following MeSH descriptors and keywords were used to ensure a comprehensive recovery of entries: ((Cancer* [tw] OR tumour* [tw] OR neoplas* [tw] OR malignan* [tw] OR carcinoma* [tw] OR metasta* [tw]) OR (“Neoplasms” [Majr] OR “neoplasms/etiology” [Mesh])) AND (("wood dust" [tw] OR "Wood dust exposure" [tw]) OR (("Wood"[Majr]) AND "Dust"[Majr:NoExp])) AND "humans"[MeSH Terms]. The Etiology/Broad filter was applied through the Clinical Queries tool. Related articles were also identified following a review of the references listed for most of the relevant works identified.

### Information selection and extraction

After the relevant studies were identified and selected, a standardized set of information was collected from each article including: the name of the authors, the year of publication, the journal of publication, the characteristics of the sample, the study design, and the result variables and their measures of association and/or impact. This procedure was carried out according to the recommendations of the PRISMA Statement [17,18].

The literature search was performed by an experienced documentalist and data extraction was performed independently by two of the authors. All discrepancies were solved by consensus.

### Scientific evidence

All selected studies were classified according to their design type, based on the classification proposed by the US Task Force and the Centre for Evidence Based Medicine (CEBM) of Oxford [19,20]. The categories included: I) Evidence obtained from a single randomized controlled trial or a meta-analysis of randomized controlled trials; IIa) Evidence obtained from at least one well-designed controlled study without randomization; IIb) Evidence obtained from at least one well-designed quasi-experimental study; III) Evidence obtained from well-designed non-experimental descriptive studies, such as comparative studies, correlation studies, and case-control studies; and IV) Evidence obtained from expert committee reports or opinions and/or clinical experience of respected authorities. The levels according to CEBM include: level 1 (systematic review of randomized control trials), level 2 (observational study with dramatic effect), level 3 (cohort study), level 4 (case-series or case-control studies), and level 5 (mechanism-based reasoning). To improve the scientific rigor of the present systematic review, all of the published articles that were classified by each of the two reviewers as level 5 were excluded.

### Meta-analysis

Case-control studies that analyzed the relationship between wood dust exposure and sinonasal ADCN were included in our meta-analysis. The following inclusion criteria were used: a) case-control studies published in peer-reviewed journals, b) description of occupational exposure to wood dust among cases and controls; and c) diagnosis of sinonasal ADCN by biopsy. The main purpose of the meta-analysis was to compare the presence of sinonasal ADCN among workers exposed to wood dust with non-exposed workers used as controls in case-control studies.

Data were extracted from the selected studies by three authors (A.-J.C, I.H.-G., and C.H.) and differences were solved by consensus. The odds ratio (OR), 95% confidence interval (CI), and *P* values are reported for the pooled results based on the use of a random effects model (DerSimonian and Laird method) [21]. A random effects model was chosen due to the low number of studies available and their observational nature. A *P*-value less than 0.05 was considered statistically significant. Cochran’s Q-statistic was used to assess heterogeneity. A significant Q-statistic value (P < 0.10) indicated heterogeneity across the studies examined. The I^2^ statistic was used to estimate inconsistency in the meta-analysis, thereby representing the percentage of the observed between-study variability due to heterogeneity rather than chance. The following suggested cut-off points were used: I^2^ = 0–25%, no heterogeneity; I^2^ = 25–50%, moderate heterogeneity; I^2^ = 50–75%, large heterogeneity; I^2^ = 75–100%, extreme heterogeneity [22].

A sensitivity analysis was performed to assess the effect of excluding individual studies in the results. The meta-analysis was performed by using the computer software package RevMan 5.0 [23].

## Results

### Systematic review

**Fig 1:**
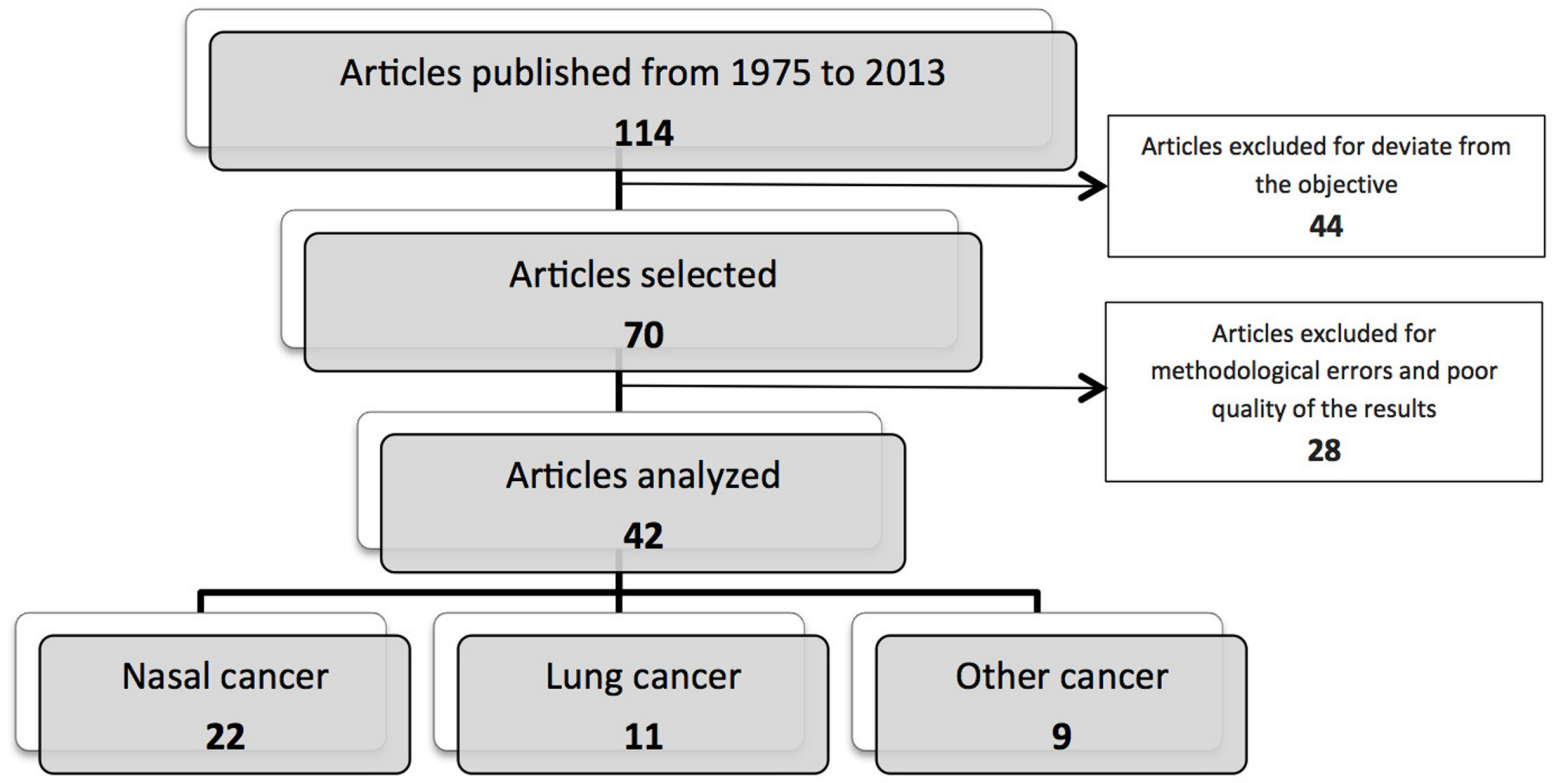
A PRISMA flowchart that illustrates the search strategy used to identify articles included in this systematic review.

Among the articles that investigated the relationship between exposure to wood dust and cancer and were published between 1975 and September 2013, 70 studies were selected for this review. Reports which only included opinions and/or recommendations from experts were excluded. Of the 70 selected studies, 42 had investigated the relationship between wood dust exposure and cancer according to the incidence of nasal ADCN (n = 22), lung cancer (n = 11), and other cancers (n = 9) (see Fig 1).

**Fig 2:**
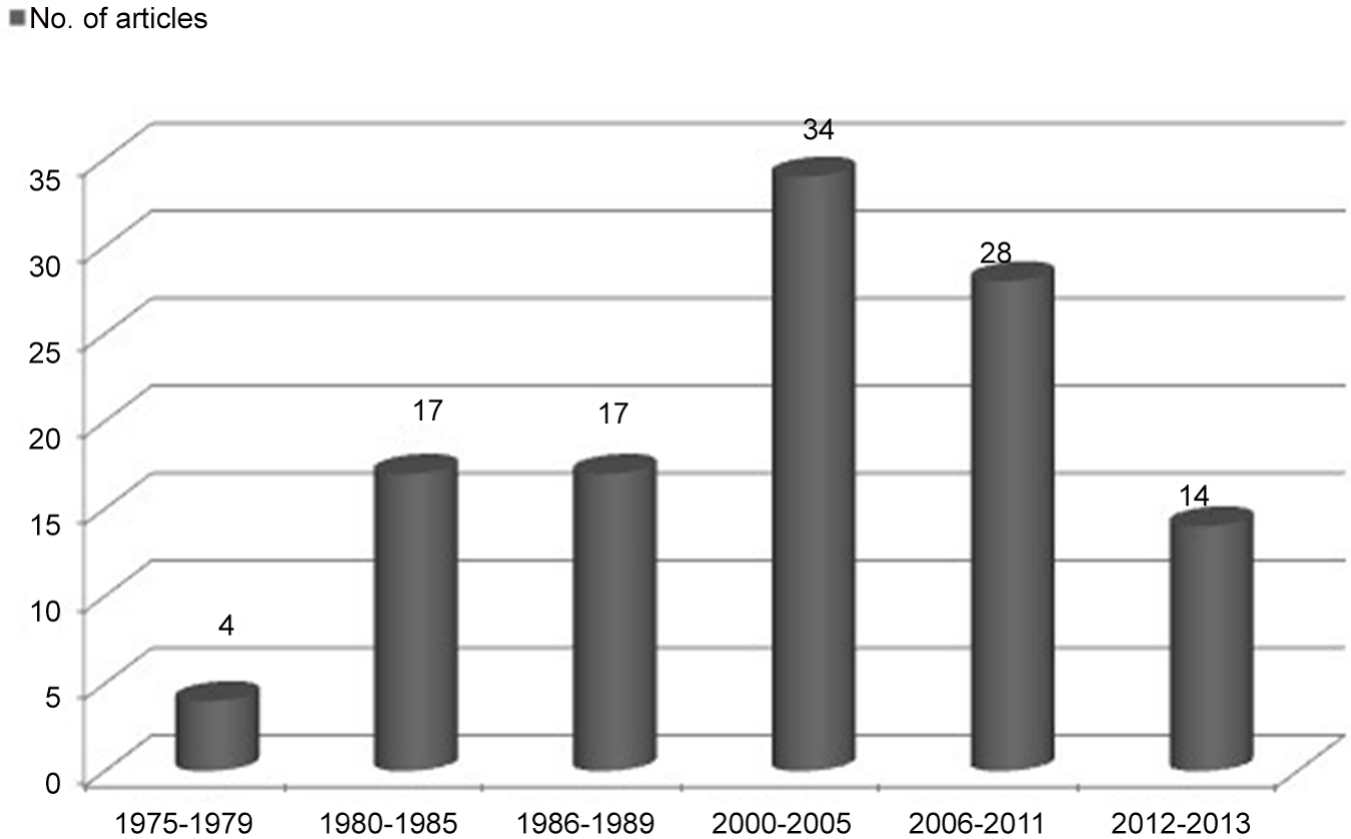
Temporal evolution of the publications related to wood dust exposure and cancer incidence that are indexed in Medline.

The historical evolution of the publications indexed in Medline is shown in Fig 2. A total of 114 articles were collected, and their chronological evolution is demonstrated in the vertical cylinders graph. As shown in Fig 2 scientific publications have progressively increased until they reached a peak between 2000 and 2013.

As shown in Table 1 most of the selected studies addressed the causal relationship of wood dust with ADCN, and case-control studies [24–45] and case series studies were predominant. However, there were other, less common study designs [27] and descriptive or population-based studies [35,38,41] as well. Furthermore, most of the studies assessed the epidemiological association by obtaining a clinical history and/or occupational history for each of the patients examined. The most widely analyzed profession was that of carpenters, followed by sawmill workers and other workers who manipulate wood in their work. OR values were the main measure of impact used in the studies. Bias control and control of confounding variables were reported in eleven studies [26,27,29–33,36,37,39–41], and these generally included research performed over the last decade. The most common risk factor that was adjusted for was tobacco consumption [26,29,30,33,41], and it was analyzed through logistic regression.

**Table 1:** Summary of published results on the relation between exposure to wood dust and nasal cancer—adenocarcinoma (ADCN).

First author, ref. no., year (in chronological order)	Quality of evidence^a/b^	No. of cases^c^	Those exposed/type of exposure	Quality indexes	Assessment of exposure	Results	Measure of association/results
**Bonzini M** ^**24**^ **, 2013**	III/4	65	Workers	No	Working exposure	Retrospective association with exposure	Higher risk with occupational exposure
**Gómez ME** ^**25**^ **. 2010**	III/4	117	Wood workers	No	Carpenter’s workshop	Association with exposure	Higher risk
**D’Errico A** ^**26**^ **,2009**	II-b/3b	113 cases of clinical incidents and 336 hospital controls	Exposure to wood, leather dust, and solvents	Yes. Adjusted for age, gender, tobacco, and other exposures	Occupational history	ADCN associated with exposure	Dose-response relationship (OR = 58)
**Pukkala E** ^**27**^ **, 2009**	II-b/2b	Cases of cancer until 2005	Occupational exposure, to wood dust	Yes. Standardized incidence ratio	Occupational history	ADCN associated with wood dust exposure	Mortality (SIR = 5.5) (IC 95%, 4.6–6.56)
**Fontana L** ^**28**^ **, 2008**	III/4	46, retrospective	Carpenters and cabinetmakers	No	Occupational history	92% ADCN after 20 years of exposure (11–27 y)	Exposure time is important
**JayapraKash V** ^**29**^ **, 2008**	II-b/3b	1522 cases of oropharyngeal cancer and 1522 controls	General population	Yes. Adjusted for other risks and tobacco	Regular exposure to wood dust for more than 20 years	Exposure increases the risk of tumors (OR = 1.32; 95% CI: 1.01–1.8)	Mildly higher risk. No OR.
**Pesch B** ^**30**^ **, 2008**	II-b/3b	86 cases, 204 controls	Wood workers, carpenters, or cabinetmakers	Yes. Adjusted for age, tobacco, and other risks	Exposure	Assessment of exposure levels, mg/m^3^	Increased risk with exposure >3.5 mg/m^3^
**Arias Bahia SH** ^**31**^ **, 2005**	III/4	138	Exposure of workers to fine wood dust	No	Occupational history	High mortality rate for tumors OR (CMOR)	Increased risk of oropharyngeal tumors
**Helmet M** ^**32**^ **, 2004**	III/4	Population study, 91 (78%) cases and 195 (75%) controls from municipal records	Wood workers	Yes. Adjusted for age and socioeconomic level	Wood sector (hard and soft)	Higher incidence in exposed workers	SIR = 1.9 (95% CI: 1.5–2.4) Soft wood SIR = 7.3 (95% CI: 1.4–22) Both SIR = 10 (95% CI: 4.7–18)
**Jansing PJ** ^**33**^ **, 2003**	III/4	28 cases of ADCN, retrospective analysis	Wood workers with exposure to hard and soft wood dust	Yes. Adjusted for other risks, tobacco, and histological type	Occupational history	No significant differences between exposure to wood types and histological types	No association between wood type and histological type of nasal cancer (epidermoid and ADCN)
**Bussi M** ^**34**^ **, 2002**	II-b/3b	68 cases and 81 volunteers	Carpenters with 10 years of exposure	No	Occupational history	Clinical protocol for early diagnosis	Increased metaplasia in nasal epithelium of exposed patients
**Luce D** ^**35**^ **, 2002**	III/4	12 studies in 7 countries	Workers without exposure	No	Occupational history	Exposure to wood dust and increased chances of developing ADCN	No available evidence
**Hildesheim A** ^**36**^ **, 2001**	II-b/3b	375 patients with ADCN and 325 controls	General population	Yes. Adjusted with LR for other risks and exposures. Blinded experiment.	Occupational history	ADCN is associated with wood dust exposure	Significant and consistent association if exposed before age 25 for more than 10 years
**t’Mannetje A** ^**37**^, **1999**	II-b/3b	Population study, cases: 104 W/451 M; controls: 241 W/1464 M	Population study	Yes	Occupational history	ADCN associated with wood dust (39%) in men, with excess risk	OR = 2.36 (95% CI: 1.7–3.2)
**Stellman SD** ^**38**^ **, 1998**	II-b/2b	Population study, Cancer Prevention Program	Wood-related workers	No	Occupational history	Small but significant excess risk	RR = 1.17 (95% CI: 1.11–1.24); RR of death = 1.17 (95% CI: 1.05–1.3)
**Leclerc A** ^**39**^ **, 1994**	II-b/3b	207 cases and 409 controls	General population	Yes. Individual assessment of each case.	Exposure to wood dust	Assessment of duration and average exposure level	Doubled risk with exposure to fine wood dust, but not with other kinds of dust
**Vaughan TL** ^**40**^ **, 1991**	II-b/3b	Studies in USA from 1979 to 1987	Population study, exposure to soft wood dust	Yes. Control of risk factors	Occupational history	Wood dust associated with increased risk	OR = 7 (95% CI: 1.4–34) for sinonasal and squamous nasopharyngeal cancer
**Hayes RB** ^**41**^ **, 1986**	II-b/3b	Population study	Wood workers	Yes. Adjusted for age and tobacco.	Several wood-related exposures	Association between ADCN and occupational activity	Wood industry (OR = 11.9) Furniture workers and cabinetmakers (OR = 39) and carpenters (OR = 16.3)
**Battista G** ^**42**^ **, 1983**	III/4	36 cases and 164 controls	Exposure to wood	No	Occupational history	ADCN associated with wood dust exposure	OR = 5.4 (95% CI: 1.7–17 for all ADCN and 87 for mucinous ADCN
**Roush GC** ^**43**^ **, 1980**	III/4	Cases and controls	Occupational exposure	No	Occupational history	ADCN associated with wood dust exposure	OR = 4 (95% CI: 1.5–10.8)
**Cecchi FA** ^**44**^ **, 1980**	III/4	69, 13 diagnosed cases	Exposure to wood and leather	No	Occupational history	ADCN associated with wood dust exposure	Significant association with occupation
**Ironside P** ^**45**^ **, 1975**	III/4	19 ADCN cases, retrospective analysis	General population	No	Clinical history and occupational exposure	More ADCN in wood workers than in general population	Significant association and different from general population

^a/b^Quality of evidence according to US Task Force on Preventive Health Care 1989 (first column)/Centre for Evidence-based Medicine, Oxford (second column).

^c^No. of ADCN cases, unless otherwise specified.

ADCN: adenocarcinoma; OR: odds ratio; RR: relative risk; SIR: standardized incidence ratio; CI: confidence interval.

Table 2 lists eleven articles that analyzed the association between exposure to wood dust and lung cancer. Of these, seven were case-control studies [29–55] and three were population and retrospective studies [48,49,53]. OR was the main measure of impact used in these articles, and bias control and control of confounding variables were only present in four studies which were conducted over the last decade [46–48,50,55]. In nine of the studies, an association between lung cancer and wood dust exposure was observed. In the other two studies, a statistically significant association was not observed, potentially due to the low quality of these studies [52,53].

**Table 2:** Summary of published results on the relation between exposure to wood dust and lung cancer.

First author, ref. no., year (in chronological order)	Quality of evidence^a/b^	No. of cases/ controls	Exposed workers	Quality indexes	Assessment of exposure	Results	Conclusion
Rake C^56^, 2009	III/4	457/792	Population study	Yes, potential confounding factors	Occupational history	Low risk for mesothelioma	OR = 4.63 (95% CI: 1.05–20.29)
Bhatti P^47^, 2011	III/4	440/845	Wood workers	No	Clinical history	Risk for sawmill workers, but not for any other workers	OR = 1.5 (95% CI: 1.1–2.1)
Fritschi L^58^, 2005	III/4	1522 cases of lung and oropharyngeal cancer/1522	Workers exposed to wood dust	No	Exposure to wood dust	Higher risk for non-Hodgkin lymphoma	OR = 1.69 (95% CI: 1.2–2.4)
Jansson C^59^, 2005	III/4	Population study	Population exposed to wood dust	No	Workers exposed to wood dust	Increased risk	SIR = 1.11 (95% CI: 1.2–11)
Briggs NC^60^, 2003	IIb/3b	1368/1192	Population study, African Americans and Mexicans	Yes, adjustments	Occupational history	Higher risk of cancer in Afro-American men	OR = 3.15 (95% CI: 1.45–6.86)
Lee WJ^61^, 2003	III/4	69/237	Construction workers	No	Occupational history	Exposure to wood dust	OR = 3 (95% CI: 0.9–4.9)
Innos K^53^, 2000	III/4	Population study, 3723 M/3063 W	Furniture factory workers	No	Occupational history	Higher risk	SIR = 1.43 (95% CI: 0.8–1.7)
Maier H^63^, 1992	III/4	199/393	Exposed workers	No	Occupational exposure	Higher risk	OR = 4.8 (95% CI: 1.2–19.0)
Wu X^55^, 1995	III/4	113 African Americans, 67 Mexican-Americans/270	Occupational exposure in ethnic groups	Yes, stratification	Occupational history	Wood dust is identified as a risk factor for African-Americans	OR = 5.5 (95% CI: 1.6–19)

^a/b^Quality of evidence according to US Task Force on Preventive Health Care 1989 (first column)/Centre for Evidence-based Medicine, Oxford (second column).

ADCN: adenocarcinoma; OR: odds ratio; RR: relative risk; SIR: standardized incidence ratio; CI: confidence interval.

The nine studies that investigated the association between exposure to wood dust and other types of cancer are listed in Table 3. Two of these articles focused on the relationship between exposure to wood dust and lymphomas [56–58,60], while the other studies involved the following types of cancer: thyroid [61–62], mesothelioma [56], multiple myeloma [61], gastric cardia [59], glottic [63], and sarcoma [57]. Case-control studies were the most common (n = 6), followed by records and case reports (n = 2) and population-based cohort studies (n = 1). Except for a study of multiple myeloma, all of the other studies identified a statistically significant association between exposure to wood dust and cancer.

**Table 3:** Summary of published results on the relationship between exposure to wood dust and other types of cancer.

First author, ref. no., year (in chronological order)	Quality of evidence^a/b^	No. of cases / controls	Exposed workers	Quality indexes	Assessment of exposure	Results	Conclusion
Rake C^56^, 2009	III/4	622 / 1420	Population study	No	Occupational history in construction (carpenters)	Low risk for mesothelioma	OR = 50.0 (95% CI: 25.8–96.8)
Merletti F^57^, 2006	III/4	Multicentric case-control study in 7 countries. 96/2632	Exposed workers	No	Clinical history, wood exposure, and exposure to other factors	Higher risk for bone sarcoma	OR = 2.68 (95% CI: 1.36–5.29)
Fritschi L^58^, 2005	IIb/3b	70/45	Total cases from 2001 and 694 controls from electoral roll	Yes	Occupational history and interview for exposure over 15–30 years.	Higher risk for non-Hodgkin lymphoma	Wood dust slightly increases risk
Jansson C^59^, 2005	IIb/2a	Population-based cohort study	Construction workers	Yes. Standardization	Workers exposed to wood dust	Higher incidence of cardia adenocarcinoma	RR = 4.8 (95% CI: 1.2–19.4)
Briggs NC^60^, 2003	IIb/3b	2073 cases of lymphoma, 612 of sarcoma/1910	African American and Caucasian workers	Yes	Occupational history of wood dust exposure	Higher risk of cancer in African American men	OR = 4.6 (95% CI: 1.6–13) for Hodgkin lymphoma and OR = 3.7 (95% CI: 1.6–8.6) for sarcomas
Lee WJ^61^, 2003	III/4	446/0	Construction workers	Yes	Occupational history	No risk of multiple myeloma associated with wood dust	RR < 1
Fincham SM^62^, 2000	III/3b	1277/2666	Workers in wood processing for pulp and papermaking	Yes	Occupational history	Higher risk of	OR = 2.5 (95% CI: 1.1–5.8) for thyroid cancer
Maier H^63^, 1992	III/4	164/656	Exposed workers	Yes	Clinical history and exposure	Higher risk of glottic cancer	RR = 3.18 (95% CI: 1.1–9.0)
Kawachi I^64^, 1989	III/4	19,904 cancer patients	Sawmill workers (S), carpenters (C), Foresters (F), and loggers (L)	No	Occupational history	Higher risk of cancer for different occupational exposures	Lung adenocarcinoma (OR = 1.76), lip ca. (OR = 2.28) and lung ca. (OR = 1.27) F/L, Nasopharyngeal ca. (OR = 6.02)

^a/b^Quality of evidence according to US Task Force on Preventive Health Care 1989 (first column)/Centre for Evidence-based Medicine, Oxford (second column).

ADCN: adenocarcinoma; OR: odds ratio; RR: relative risk; CI: confidence interval.

Table 4 lists the reviews that have been published regarding exposure to wood dust and cancer. The list includes a meta-analysis [64–65] of larynx cancer, two systematic reviews [66–69], and five narrative reviews [33,67,68,70,71]. In the former, it was concluded that there was no association between larynx cancer and wood dust exposure.

**Table 4:** Summary of systematic reviews and meta-analyses of exposure to wood dust.

First author, ref. no., year, type of study	Quality of evidence^a/b^	No. of studies included	Objective	Workers	Conclusions
Paget-Bailly S^65^, 2012. Meta-analysis	II-b/3a	10 studies with homogeneous exposure	Incidence of laryngeal cancer	Different types of occupational exposure (wood dust)	Risk is not significantly associated with workers exposed to wood dust
Puñal-Riobóo J^66^, 2010. Systematic review	II-b/3b	10 cases and controls selected by 2 researchers	Association between occupational exposure to substances and cancer	Occupational exposure. Statistical association between cancer and exposure	Exposure is associated with a higher risk of nasopharyngeal and hypopharyngeal cancers. There are non-concurrent data
De Gabory L^67^, 2009. Narrative review	III/5	Classic review that assesses scientific evidence	Association between ADCN and wood dust	Risk is important from the first year. When the period of exposure is > 30 y, only 10% of patients are < 50-years-old	Exposure to wood dust plays an essential role in the development of nasal ADCN
Jansing PJ^33^, 2003. Narrative review	III/4	Retrospective study of 28 patients with nasal cancer	Profiles of different risk factors, occupational and non-occupational	Risk is important from the first year, and the period is generally > 30 y, only 10% of patients are < 50-years-old	Preventive activities are recommended
Blot WJ^68^, 1997. Narrative review	III/5	Population study, USA and EU	Assessment of occupational history	Wood workers	Threshold dose equivalent to 8 h at 5 mg/m^3^
Demers PA^69^, 1995. Systematic review	III/3b	Review of 12 studies in 7 countries	Risk of nasal cancer	OR = 45.5 (95% CI: 28.3–72.9). Risk increases according to the time of occupational exposure	Results increase consistency of individual studies between ADCN and wood dust
Nylander LA^70^, 1993. Narrative review	III/5	Opinion from experts	Risk of nasal cancer	Higher risk of occupational cancer	No data or direct experimental evidence for the dangers of wood dust
Wills H^71^, 1982. Narrative review	III/4	Register data from 12 countries	Population study	Higher risk of occupational cancer	61% of neoplasms of the respiratory tract and 78% of ADCNs are associated with furniture manufacture or manipulation

EU: European Union; CI: confidence interval; ADCN: adenocarcinoma.

^a/b^Type of epidemiological design determined according to US Task Force on Preventive Health Care 1989/Centre for Evidence-based Medicine, Oxford.

### Meta-analysis

**Fig 3:**
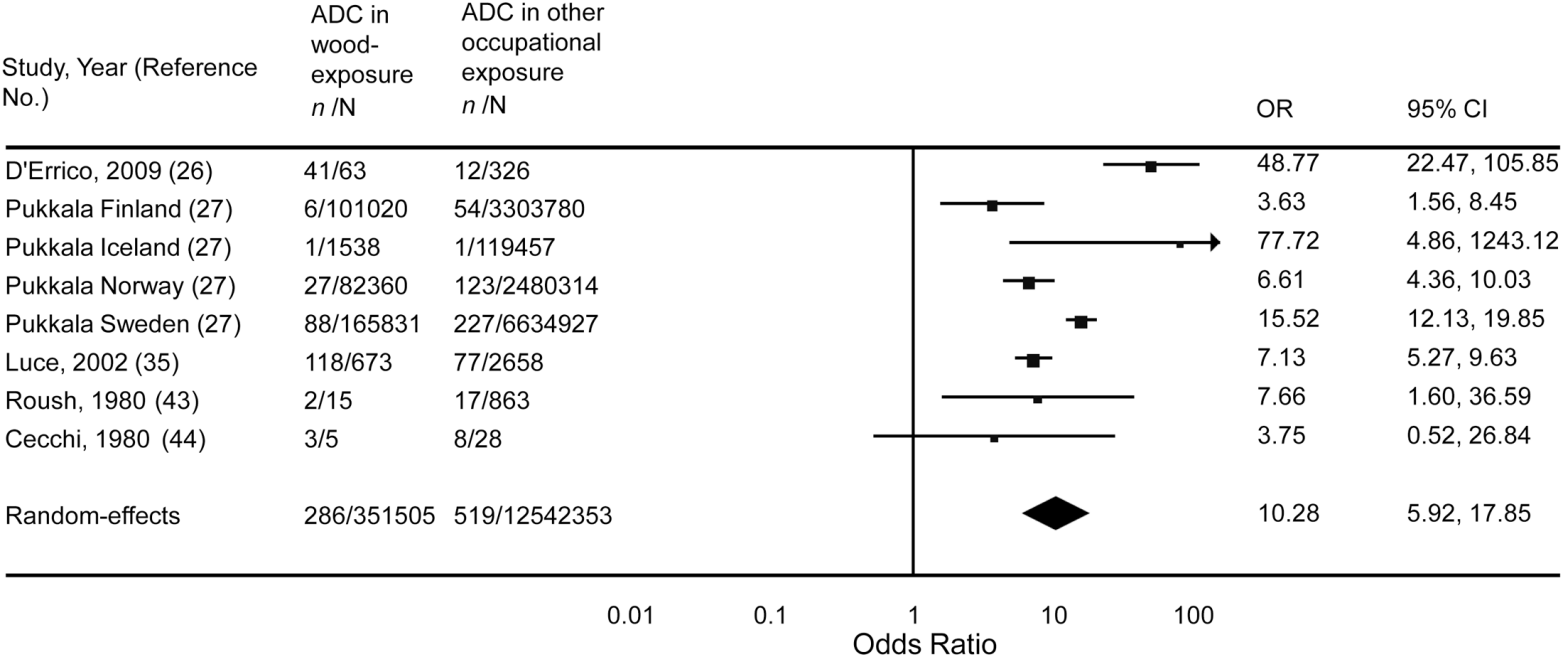
Meta-analysis of the association of sinonasal ADCN with wood exposure. Labor-based wood exposure was compared with other occupational exposures. Test for overall effect: Z = 8.28 (*P* <0.0001). Test for heterogeneity: χ^2^ = 46.17 (*P* <0.0001), I^2^ = 85%.

Among the studies included in our systematic review, and according to the predefined criteria for this study, five reports were selected for a meta-analysis [26,35,43,44]. The summary OR under the random-effects model showed that subjects with wood dust exposure exhibited higher rates of sinonasal ADCN compared with non-exposed workers (see Fig 3: OR = 10.28; 95% CI: 5.92 and 17.85, respectively; P<0,0001). A large degree of heterogeneity was also observed between the studies (I^2^ = 85%). However, in the sensitivity analysis, the exclusion of individual studies did not change this significant result (**see** Table 5).

**Table 5:** Patients with ADCN according to their occupational exposure from studies included in the meta-analysis.

First author, ref. no., year	Criteria selection	ADCN among those exposed to wood dust			ADCN with other		
		Events	Controls	Total	Events	Controls	Total
d'Errico et al^26^, 2009	OE: Information was collected regarding lifetime occupational exposure	41	22	63	12	314	326
Pukkala et al^27^, 2009		122	350627	350749	405	12538073	12538478
-Finland substudy	OE: Obtained from the national adaptations of the Nordic Occupational Classification	6	101014	101020	54	3303726	3303780
-Iceland substudy	OE: Obtained and converted from the International Standard Classification of Occupations from 1968 (ISCO-68)	1	1537	1538	1	119456	119457
-Norway substudy	OE: Obtained from the national adaptations of the Nordic Occupational Classification	27	82333	82360	123	2480191	2480314
-Sweden substudy	OE: Obtained from the national adaptations of the Nordic Occupational Classification	88	165743	165831	227	6634700	6634927
Luce et al^35^, 2002	OE: Detailed information on occupational history was collected in all studies, including job description and years of employment for each job held	118	555	673	77	2581	2658
Roush et al^43^, 1980	OE: Obtained from death certificates and directories from occupational information	2	13	15	17	846	863
Cecchi et al^44^, 1980	OE: Occupational history data	3	2	5	8	20	28

ADCN: Adenocarcinoma.; OE: occupational exposure.

## Discussion

The studies that were selected and reviewed show that: 1) wood dust may act as a carcinogen, and 2) there is an association between exposure to wood dust and nasal ADCN, and to a lesser extent, with lung cancer. The results of several studies also suggest that there is a relationship between exposure to wood dust and other types of cancer, although there is currently not sufficient data or evidence to clearly establish this association. Moreover, the design of the included studies (mostly case-control studies) potentially limits the strength of the latter association.

The historical evolution of studies in this field has grown in parallel with the interest and impact of wood dust as an occupational exposure. At first, epidemiological research was mainly observational, and was based on case studies that involved occupations where exposure to wood dust was a factor. Thus, the early studies were of carpenters and sawmill workers. However later on, the studies included other situations where wood is manipulated or processed as a secondary and/or complementary activity. Analytical case-control studies also started to be conducted, thereby adding scientific rigor to the hypothesis by establishing comparisons with healthy controls. The addition of multicentric studies provided further strength to the observed associations.

Most of the evidence presented in the present study was extracted from case-control analytical observational studies. This type of study design, as pointed out in two previous systematic reviews [66,69], is the most appropriate since it makes it possible to study diseases with a large latency period and to assess several types of exposure concomitantly. This study design is also economical and rapid. However, case-control studies do have limitations. For example, potential bias exists in the selection of controls, the temporal relationship between the presumed cause and the studied effect cannot be determined, and there is a need for specific biases to be controlled through adjustment techniques. These considerations have been highlighted in most of the studies and works published over the last two decades. However, advances in statistical techniques or procedures have made it possible to control confounding factors and biases, thus improving the quality of the observations.

Overall, a positive evolution in the methodological quality of the studies and research on wood dust exposure has lead to the publication of evidence that has greater validity and reliability. There was also a wide geographical distribution for the studies that were considered, with the majority conducted in Europe and the United States, followed by studies conducted in Brazil and Colombia. The first study was conducted in 1975 in Australia [45]. However, very few of these studies were included in our meta-analysis.

Apart from the relationship between wood dust and nasal ADCN, the association between exposure to wood dust and other neoplasms, such as lung cancer, has also been assessed. Based on the data of the studies published to date, this association has not been established. Primarily due to the low quality of the evidence collected, the association has been weakly demonstrated in most studies, and evidence was nonexistent in two other studies [52,53]. Consequently, while a potential association exists, it remains to be validated.

### Limitations

It is possible that certain factors in each research or review process can alter the final results. The main limitation of the results of the present review derives from the type of study design that was employed in most of the studies conducted. For example, most of the evidence available is from observational studies, and specifically, from analytical case-control studies. Consequently, a temporal relationship between wood dust exposure and cancer cannot be determined. We also acknowledge that the use of other databases apart from PubMed could have yielded additional results, although we hypothesize that the final result of our work would be similar.

The most important limitation of the articles that were analyzed in this systematic review is that the characterization of occupational exposure in many of the articles published, particularly up until the 1990s, has been retrospective. In addition, the characterization was assessed through the working and occupational history of the patients examined. The latter aspect may be further biased by the quality of the documents examined, the maintenance of the working and occupational history files, and the memory of the workers. Other important limitations include the low number of studies with a cohort-type or prospective monitoring design, the sample size of individual studies, and the existence of few multicentric studies; although the latter is diminished by the wide geographic distribution of the studies that have been conducted. Another limitation of the earlier studies is the lack of adjustment for occupational or environmental risk factors that can act as confounders. These should be adjusted and/or neutralized since they can be linked to both the exposure (cause) and the effect; with a specific example being tobacco.

Regarding the meta-analysis performed, there were very few studies that met the inclusion criteria due to methodological differences. In addition, large heterogeneity was observed between the included studies. Therefore, although our meta-analysis confirms that a significant relationship between wood dust and nasal ADCN exists, this result should be approached with caution.

## Conclusions

The conclusion of this systematic review is that there is low-to-moderate quality evidence that supports a causal association between the incidence of cancer and occupational exposure to wood dust. However, the association between exposure to wood dust and nasal ADCN is stronger, largely because most of the causal criteria established by Bradford Hill have been assessed [72]. In regard to lung cancer, caution is still advised in establishing an association with wood dust exposure given the low number of studies that have been conducted and their poor methodological quality.

Nevertheless, it is apparent that there is a need to implement preventive measures for workers exposed to wood dust. We propose that it is appropriate and adequate to establish a series of primary and secondary preventive measures in professional and working environments in order to improve the working health, hygiene, and safety of workers exposed to wood dust. It is critical that future studies overcome the limitations that have been observed in the present systematic review, particularly by identifying the characteristics of occupational exposure, adjusting for other exposures and confounding factors, increasing the sample size, and making comparisons with better control groups.

## Supporting Information

S1 File PRISMA checklist. (DOC)

S2 File Supplementary Methods. (DOCX)

## References

[1] Labrèche F, Duguay P, Ostiguy C, Boucher A, Roberge B, Peters CE, et al Estimating occupational exposure to carcinogens in Quebec. Am J Ind Med. 2013 9;56 (9):1040–50. 10.1002/ajim.22200 23804516

[2] Hinds WC. Basis for particle size-selective sampling for wood dust. Applied Industrial Hygiene 1988;3:67–72.

[3] Scheeper B, Kromhout H, Boleij JS. Wood-dust exposure during wood-working processes. Ann Occup Hyg 1995;39:141–54. 7741413

[4] Teschke K, Demers PA, Davies HW, Kennedy SM, Marion SA, Leung V. Determinants of exposure to inhalable particulate, wood dust, resin acids, and monoterpenes in a lumber mill environment. Ann Occup Hyg 1999;43:247–55. 10432869

[5] Celik A, Kanik A. Genotoxicity of occupational exposure to wood dust: Micronucleus frequency and nuclear changes in exfoliated buccal mucosa cells. Environ Mol Mutagen 2006;47:693–8. 17078100 10.1002/em.20257

[6] Macbeth R. Malignant Disease of the Paranasal Sinuses. J Laryngol Otol 1965;79:592–612. 14335138 10.1017/s0022215100064112

[7] Acheson ED, Cowdell RH, Hadfield E, Macbeth RG. Nasal cancer in woodworkers in the furniture industry. Br Med J 1968;2:587–96. 5654629 10.1136/bmj.2.5605.587 PMC1991769

[8] Andersen HC, Andersen I, Solgaard J. Nasal cancers, symptoms and upper airway function in woodworkers. Br J Ind Med 1977;34:201–7. 911690 10.1136/oem.34.3.201 PMC1008231

[9] Brinton LA, Blot WJ, Becker JA, Winn DM, Browder JP, Farmer JC Jr, et al A case-control study of cancers of the nasal cavity and paranasal sinuses. Am J Epidemiol 1984;119:896–906. 6731431 10.1093/oxfordjournals.aje.a113812

[10] Demers PA, Kogevinas M, Boffetta P, Leclerc A, Luce D, Gérin M, et al Wood dust and sino-nasal cancer: pooled reanalysis of twelve case-control studies. Am J Ind Med 1995;28:151–66. 8585514 10.1002/ajim.4700280202

[11] Demers PA, Boffetta P, Kogevinas M, Blair A, Miller BA, Robinson CF, et al Pooled reanalysis of cancer mortality among five cohorts of workers in wood-related industries. Scand J Work Environ Health 1995;21:179–90. 7481605 10.5271/sjweh.26

[12] Carosso A, Ruffino C, Bugiani M. Respiratory diseases in wood workers. Br J Ind Med 1987;44:53–6. 3814535 10.1136/oem.44.1.53 PMC1007778

[13] Barcenas CH, Delclos GL, El-Zein R, Tortolero-Luna G, Whitehead LW, Spitz MR. Wood dust exposure and the association with lung cancer risk. Am J Ind Med 2005;47:349–57. 15776474 10.1002/ajim.20137

[14] IARC Working Group on the Evaluation of Carcinogenic Risks to Humans., International Agency for Research on Cancer. Wood dust and formaldehyde. Lyon: World Health Organization, International Agency for Research on Cancer; 1995.

[15] Chamorro AJ, Marcos M, Mirón-Canelo JA, Cervera R, Espinosa G. Val247Leu Beta2-glycoprotein-I allelic variant is associated with antiphospholipid syndrome: systematic review and meta-analysis. Autoinmunity Reviews 2012; 11: 705–12. 10.1016/j.autrev.2011.12.006 22246055

[16] Chamorro AJ, Marcos M, Mirón-Canelo JA, Pastor I, González-Sarmiento R, Laso FJ. Association of μ-opioid receptor (OPRM1) gene polymorphism with response to naltrexone in alcohol dependence: a systematic review and meta-analysis. Addict Biol. 2012;17:505–12 10.1111/j.1369-1600.2012.00442.x 22515274

[17] Vandenbroucke JP, von Elm E, Altman DG, Gøtzsche PC, Mulrow CD, Pocock SJ, et al Strengthening the Reporting of Observacional Studies in Epidemiology (STROBE): explanation and elaboration. Ann Intern Med 2007; 147: 163–94. 10.7326/0003-4819-147-8-200710160-00010-w1 17938389

[18] Urrútia G, Bonfill X. PRISMA declaration: A proposal to improve the publication of systematic reviews and meta-analyses. Med Clin (Barc) 2010; 135:507–511. 20206945 10.1016/j.medcli.2010.01.015

[19] US Preventive Task Force. Guide to Clinical Preventive services: an assessment to the effectiveness of 169 interventions. Baltimore: Wiliams and Wilkins, 1989.

[20] Centre for Evidence-based Medicine (CEBM). Levels of Evidence (March 2009). Oxford University: CEBM, 2013. Available: http://www.cebm.net/?o=1025.

[21] Cochran WG. The combination of estimates from different experiments. Biometrics 1954; 10: 101–29.

[22] Higgins JP, Thompson SG, Deeks JJ, Altman DG. Measuring inconsistency in meta-analyses. BMJ 2003; 327: 557–60. 12958120 10.1136/bmj.327.7414.557 PMC192859

[23] Review Manager (RevMan) [computer program]. Version 5.0. Copenhagen: The Nordic Cochrane Centre, The Cochrane Collaboration; 2008.

[24] Bonzini M, Battaglia P, Parassoni D, Casa M, Facchinetti N, Turri-Zanoni M, et al Prevalence of occupational hazards in patients with different types of epithelial sinonasal cancers. Rhinology. 2013; 51:31–6. 10.4193/Rhino11.228 23441309

[25] Gómez ME, Sánchez JF, Cardona AM, Pioquinto JF, Torres P, Sanchez D, et al Health and working conditions in carpenter's workshops in Armenia (Colombia). Ind Health. 2010; 48:222–30. 20424355 10.2486/indhealth.48.222

[26] D'Errico A, Pasian S, Baratti A, Zanelli R, Alfonzo S, Gilardi L, et al A case-control study on occupational risk factors for sino-nasal cancer. Occup Environ Med. 2009; 66:448–55. 10.1136/oem.2008.041277 19153109 PMC2693673

[27] Pukkala EI, Martinsen JI, Lynge E, Gunnarsdottir HK, Sparén P, Tryggvadottir L, et al Occupation and cancer—follow-up of 15 million people in five Nordic countries. Acta Oncol. 2009;48:646–790. 10.1080/02841860902913546 19925375

[28] Fontana L, Liétin B, Catilina P, Devif C, Féneon B, Martin F, et al Occupational exposure to wood dust and nasal sinus cancer. Ann Otolaryngol Chir Cervicofac. 2008; 125:65–71. 10.1016/j.aorl.2007.10.003 18436189

[29] Jayaprakash V, Natarajan KK, Moysich KB, Rigual NR, Ramnath N, Natarajan N, et al Wood dust exposure and the risk of upper aero-digestive and respiratory cancers in males. Occup Environ Med. 2008;65:647–54. 10.1136/oem.2007.036210 18182588

[30] Pesch B, Pierl CB, Gebel M, Gross I, Becker D, Johnen G, et al Occupational risks for adenocarcinoma of the nasal cavity and paranasal sinuses in the German wood industry. Occup Environ Med. 2008; 65:191–6. 17881467 10.1136/oem.2007.033886

[31] Arias-Bahia SH, Echenique Mattos I, Koifman S. Cancer and wood-related occupational exposure in the Amazon region of Brazil. Environ Res. 2005; 99:132–40. 16053937 10.1016/j.envres.2004.12.005

[32] Helmet M, Graström C, Hemminki K. Occupational risks for nasal cancer in Sweden. J Occup Environ Med. 2004; 46:1033–40. 15602177 10.1097/01.jom.0000141653.30337.82

[33] Jansing PJ, Chanda R, Gore C, Küpper T. Profiles of occupational exposure in patients with wood dust-induced nasal carcinoma. Int J Occup Med Environ Health. 2003; 16:329–35. 14964642

[34] Bussi M, Gervasio CF, Riontino E, Valente G, Ferrari L, Pira E, et al Study of ethmoidal mucosa in a population at occupational high risk of sinonasal adenocarcinoma. Acta Otolaryngol 2002;122:197–201. 11936913 10.1080/00016480252814225

[35] Luce D, Leclerc A, Bégin D, Demers PA, Gérin M, Orlowski E, et al Sinonasal cancer and occupational exposures: a pooled analysis of 12 case-control studies. Cancer Causes Control. 2002;13:147–57. 11936821 10.1023/a:1014350004255

[36] Hildesheim A, Dosemeci M, Chan CC, Chen CJ, Cheng YJ, Hsu MM, et al Occupational exposure to wood, formaldehyde, and solvents and risk of nasopharyngeal carcinoma. Cancer Epidemiol Biomarkers Prev. 2001; 10:1145–53. 11700262

[37] 't Mannetje A, Kogevinas M, Luce D, Demers PA, Bégin D, Bolm-Audorff U, et al Sinonasal cancer, occupation, and tobacco smoking in European women and men. Am J Ind Med. 1999; 36:101–7. 10361593 10.1002/(sici)1097-0274(199907)36:1<101::aid-ajim14>3.0.co;2-a

[38] Stellman SD, Demers PA, Colin D, Boffetta P. Cancer mortality and wood dust exposure among participants in the American Cancer Society Cancer Prevention Study-II (CPS-II). Am J Ind Med. 1998;34:229–37. 9698991 10.1002/(sici)1097-0274(199809)34:3<229::aid-ajim4>3.0.co;2-q

[39] Leclerc A, Martinez Cortes M, Gérin M, Luce D, Brugère J. Sinonasal cancer and wood dust exposure: results from a case-control study. Am J Epidemiol. 1994; 140:340–9. 8059769 10.1093/oxfordjournals.aje.a117256

[40] Vaughan TL, Davis S. Wood dust exposure and squamous cell cancers of the upper respiratory tract. Am J Epidemiol 1991; 15;133:560–4. 2006642 10.1093/oxfordjournals.aje.a115927

[41] Hayes RB, Gerin M, Raatgever JW, de Bruyn A. Wood-related occupations, wood dust exposure, and sinonasal cancer. Am J Epidemiol. 1986;124:569–77. 3752051 10.1093/oxfordjournals.aje.a114429

[42] Battista G, Cavallucci F, Comba P, Quercia A, Vindigni C, Sartorelli E. A case-referent study on nasal cancer and exposure to wood dust in the province of Siena, Italy. Scand J Work Environ Health. 1983; 9:25–9. 6857185 10.5271/sjweh.2446

[43] Roush GC, Meigs JW, Kelly JA, Flannery JT, Burdo H. Sinonasal cancer and occupation: a case-control study. Am J Epidemiol. 1980;111:183–93. 7355881 10.1093/oxfordjournals.aje.a112886

[44] Cecchi F, Buiatti E, Kriebel D, Nastasi L, Santucci M. Adenocarcinoma of the nose and paranasal sinuses in shoemakers and woodworkers in the province of Florence, Italy (1963–77). Br J Ind Med 1980;37:222–5. 7426471 10.1136/oem.37.3.222 PMC1008698

[45] Ironside P, Matthews J. Adenocarcinoma of the nose and paranasal sinuses in woodworkers in the state of Victoria, Australia. Cancer. 1975;36:1115–24. 1182665 10.1002/1097-0142(197509)36:3<1115::aid-cncr2820360342>3.0.co;2-v

[46] Corbin M, McLean D, Mannetje A, Dryson E, Walls C, McKenzie F, et al Lung cancer and occupation: A New Zealand cancer registry-based case-control study. Am J Ind Med. 2011;54:89–101. 10.1002/ajim.20906 20957667

[47] Bhatti P, Newcomer L, Onstad L, Teschke K, Camp J, Morgan M, et al Wood dust exposure and risk of lung cancer. Occup Environ Med. 2011;68:599–604. 10.1136/oem.2010.060004 21071755 PMC3184400

[48] Pronk A, Coble J, Ji BT, Shu XO, Rothman N, Yang G, et al Occupational risk of lung cancer among lifetime non-smoking women in Shanghai, China. Occup Environ Med. 2009; 66:672–8. 10.1136/oem.2008.043695 19625285 PMC3007593

[49] Laakkonen A, Kyyronen P, Kauppinen T, Pukkala EI. Occupational exposure to eight organic dusts and respiratory cancer among Finns. Occup Environ Med. 2006; 63:726–33. 16601013 10.1136/oem.2005.025825 PMC2077994

[50] Barcenas CH, Delclos GL, El-Zein R, Tortolero-Luna G, Whitehead LW, Spitz MR. Wood Dust exposure and the association with lung cancer risk. Am J Ind Med. 2005; 47:349–57. 15776474 10.1002/ajim.20137

[51] Dement J, Pompeii L, lipkus IM, Samsa GP. Cancer incidence among union carpenters in New Jersey. J Occup Environ Med 2003; 45:1059–67. 14534447 10.1097/01.jom.0000085892.01486.6a

[52] Szadkowska-Stanczyk I, Szymczak W. Nested case-control study of lung cancer among pulp and paper workers in relation to exposure to dust. Am J Ind Med 2001;39:547–56. 11385638 10.1002/ajim.1053

[53] Innos K, Rahu M, Rahu K, Lang I, Leon DA. Wood dust exposure and cancer incidence: a retrospective cohort study of furniture workers in Estonia. A J of Industrial Medicine. 2000: 37:501–11. 10.1002/(sici)1097-0274(200005)37:5<501::aid-ajim6>3.0.co;2-t 10723044

[54] Matos EL, Vilensky M, Mirabelli D, Boffetta P. Occupational exposures and lung cancer in Buenos Aires, Argentina. J Occup Environ Med. 2000; 42:653–9. 10874659 10.1097/00043764-200006000-00017

[55] Wu X, Delclos GL, Annegers JF, Bondy ML, Honn SE, Henry B, et al A case-control study of wood dust exposure, mutagen sensitivity, and lung cancer risk. Cancer Epidemiol Biomarkers Prev. 1995; 4:583–8. 8547823

[56] Rake C, Gilham C, Haltch J, Darnton A, Hodgson J, Peto J. Occupational, domestic and environmental mesothelioma risks in the British population: a case-control study. British Journal of Cancer. 2009; 100:1175–83. 10.1038/sj.bjc.6604879 19259084 PMC2669989

[57] Merletti F, Richiardi L, Bertoni F, Ahrens W, Buemi A, Costa-Santos C, et al Occupational factors and risk of adult bone sarcomas: a multicentric case-control study in Europe. Int J Cancer. 2006 1;118:721–7 16108052 10.1002/ijc.21388

[58] Fritschi L, Benke G, Hughes AM, Kricker A, Vajdic CM, Grulich A, et al Risk of non-Hodgkin lymphoma associated with occupational exposure to solvents, metals, organic dusts and PCBs (Australia). Cancer Causes Control. 2005; 16:599–607. 15986116 10.1007/s10552-004-7845-0

[59] Jansson C, Johansson AL, Bergdahl IA, Dickman PW, Plato N, Adami J, et al Occupational exposures and risk of esophageal and gastric cardia cancers among male Swedish construction workers. Cancer Causes Control. 2005; 16:755–64. 16049815 10.1007/s10552-005-1723-2

[60] Briggs NC, Levine RS, Hall HI, Cosby O, Brann EA, Hennekens CH. Occupational risk factors for selected cancers among African American and White men in the United States. Am J Public Health. 2003;93:1748–52. 14534232 10.2105/ajph.93.10.1748 PMC1448044

[61] Lee WJ, Baris D, Järvholm B, Silverman DT, Bergdahl IA, Blair A. Multiple myeloma and diesel and other occupational exposures in swedish construction workers. Int J Cancer. 2003 20; 107:134–8. 12925968 10.1002/ijc.11351

[62] Fincham SM, Ugnat AM, Hill GB, Kreiger N, Mao Y. Is occupation a risk factor for thyroid cancer? Canadian Cancer Registries Epidemiology Research Group. J Occup Environ Med. 2000;42:318–22. 10738710 10.1097/00043764-200003000-00013

[63] Maier H, Gewelke U, Dietz A, Thamm H, Heller WD, Weidauer H. Laryngeal cancer and occupation—results of the Heidelberg laryngeal cancer study. HNO. 1992; 40:44–51. 1568886

[64] Kawachi I, Pearce N, Fraser J. A New Zealand Cancer Registry-based study of cancer in wood workers. Cancer 1989;64:2609–13. 2819671 10.1002/1097-0142(19891215)64:12<2609::aid-cncr2820641234>3.0.co;2-e

[65] Paget-Bailly S, Cyr D, Luce D. Occupational exposures and cancer of the larynx-systematic review and meta-analysis. J Occup Environ Med. 2012; 54:71–84. 10.1097/JOM.0b013e31823c1343 22157731

[66] Puñal-Riobóo J, Varela-Lema L, Barros-Dios JM, Juiz-Crespo MA, Ruano-Raviña A. Occupation as a risk factor for oral and pharyngeal cancer. Acta Otorrinolaringol Esp. 2010;61:375–83. 10.1016/j.otorri.2009.03.009 19850270

[67] De Gabory L, Conso F, Barry B, Stoll D. Carcinogenesis of the ethmoidal adenocarcinoma due to wood dust. Rev Laryngol Otol Rhinol (Bord). 2009; 130:93–104. 19813471

[68] Blot WJ, Chow WH, McLaughlin JK. Wood dust and nasal cancer risk. A review of the evidence from North America. J Occup Environ Med. 1997;39:148–56. 9048321 10.1097/00043764-199702000-00012

[69] Demers PA, Kogevinas M, Boffetta P, Leclerc A, Luce D, Gérin M, et al Wood dust and sino-nasal cancer: pooled reanalysis of twelve case-control studies. Am J Ind Med. 1995;28:151–66. 8585514 10.1002/ajim.4700280202

[70] Nylander LA, Dement JM. Carcinogenic effects of wood dust: review and discussion. Am J Ind Med. 1993; 5: 619–647. 10.1002/ajim.4700240511 8266936

[71] Wills JH. Nasal carcinoma in woodworkers: a review. J Occup Med. 1982;24:526–30. 6750057

[72] Schünemann H, Hill S, Guyatt G, Akl EA, Ahmed F. The GRADE approach and Bradford Hill's criteria for causation. J Epidemiol Community Health. 2011;65:392–5. 10.1136/jech.2010.119933 20947872

