# Supplementary material for: Association between Occupational Exposure to Wood Dust and Cancer: A Systematic Review and Meta-Analysis
Source: PLoS One. 2015 Jul 20;10(7):e0133024. doi: 10.1371/journal.pone.0133024 (PMC4507857; doi:10.1371/journal.pone.0133024)
Supplement: S2 File — (DOCX) [file pone.0133024.s002.docx]

**SUPPLEMENTARY METHODS**

The search strategy was as follows:

1. Cancer* [tw] OR tumour* [tw] OR neoplas* [tw] OR malignan* [tw] OR carcinoma* [tw] OR metasta* [tw]

2. “Neoplasms” [Majr] OR “neoplasms/etiology” [Mesh]

*3. #1 OR #2*

*4. "wood dust" [tw] OR "Wood dust exposure" [tw]*

5. ("Wood"[Majr]) AND "Dust"[Majr:NoExp]

6. #4 OR #5

7. #3 AND #6

8. # 3 AND #6 AND "humans"[MeSH Terms]

The filter *Etiology/Broad* was applied through the *Clinical Queries* tool.
